# The melanocyte photosensory system in the human skin

**DOI:** 10.1186/2193-1801-2-158

**Published:** 2013-04-12

**Authors:** Bhanu Iyengar

**Affiliations:** Pigment Cell Centre, Iyengar Farm, Brijwasan Road, PO Kapshera, New Delhi, 110037 India

**Keywords:** Photoreceptor, Photoperiod, Circadian, Photosensitive enzymes, Seasonal light cycles, Placodal system

## Abstract

The pigment cells form the largest population of neural crest cells to migrate into the epidermis and hair follicle along each dermatomic area from the neural folds. The melanopsin system responsible for photoentrainment, was isolated from the photosensitive dermal melanophores of frogs *Xenopus laevis* responding to light. Melanocytes form a photoresponsive network which reads the environmental seasonal variations in the light cycles in the same manner. The present work was undertaken to study the organization of this system by: I. Experimental assessment of photoresponse and II. Evidence of an organized system of photoreception in the skin. Melanocytes, in whole skin organ cultures and epidermal strips, from margin of vitiligo in G_2_ phase show prominent dendricity, and express pigment, biogenic amines and hormones on UV exposure. The photoresponse depends on the photosensitive enzymes NAT/HIOMT and dopaoxidase. Melanocytes interact with adjacent keratinocytes, dermal capillaries, and nerve endings. The melanocyte network reads the diurnal and seasonal photophase by the melatonin/serotonin switch like the pineal. Sleep disorders and winter depression are corrected by phototherapy utilising this mechanism. Melanocytes showing photoactivity, aplasia, hypoplasia and hyperplasia, and interactive keratinocytes occupy the trigeminal, brachial and lumbosacral dermatomes, zones of high embryonic induction, forming an ectodermal placodal system. Melanin units and hair follicles serve as photoreceptors. Migration of active melanocytes to defined areas is evident in pigment patterns in guinea pigs. This study identifies defined photoreceptor melanocyte/epidermal domains which read the seasonal photophase and control the sleep waking cycle in response to the environmental light. I. Whole skin organ cultures, and epidermal strips from margin of vitiligo in G_2_ phase are exposed to UV and IR to study sequential and dose response of marginal melanocytes, using histochemistry, immunohistochemistry to assess pigment, biogenic amines and hormones on UV exposure. II. Dermatomic Distributions: Detailed maps of melanocyte photoresponse in 356 biopsies, lesions in 297 vitiligo, 100 melanosis, 165 melanomas 142 leprosy and 442 basal cell/keratinocytes lesions were assessed for patterns of dermatomic distribution. Embryonal melanocyte migration along dermatomes was assessed in 285 guinea pigs from an inbred colony having black, brown and white patches.

**Electronic supplementary material:**

The online version of this article (doi:10.1186/2193-1801-2-158) contains supplementary material, which is available to authorized users.

## Background

The pigment cells form the largest population of neural crest cells to migrate into the epidermis and hair follicle along each dermatomic area (Williams et al. 1995) from the neural folds. The melanocytes respond to sunlight with tanning (Fitzpatrick et al. 1983) and camouflage in lower animals (Buckmann 1985). The melanopsin system of mammals, expressed in a small subset of retinal ganglion cells, regulating and adjusting circadian rhythms, to the environmental light/dark cycle, known as photoentrainment, was in fact isolated from the photosensitive dermal melanophores of frogs *Xenopus laevis* responding to light (Daniolos et al. 1990; Provencio et al. 1998, 2000). Extensive studies on the melanocytes by the present author reveal that these fascinating and versatile cells form a photoresponsive network which reads the environmental seasonal variations in the light cycles (Iyengar [Bibr CR23][Bibr CR24], 1994, 1996a, [Bibr CR32][Bibr CR33][Bibr CR34], 2000) in the same manner. The present work was undertaken to study the organization of this system in the human skin. The work is presented in two sections:

I. Experimental assessment of photoresponse and
II. Evidence of an organized system of photoreception in the skin.

## Results

### Experimental assessment of photoresponse

All observations in this study were made on whole skin organ cultures taken from margins of vitiliginous skin to include the margin between the depigmented area, with absence of melanocytes, and the marginal pigmented zone containing inactive melanocytes. The amelanotic zone serves as the control while the quiescent marginal melanocytes can be experimentally activated or inactivated to study various functions. Five marginal melanin units [MU] are assessed in each section since these are the reactive melanocytes. Flat mounts of epidermal strips were included to get a 3D impression of the photoresponse. All hair follicles in OCs have been studied for melanocyte photoresponse.

### Photoresponsive melanocytes: [Figure 1]

**Figure 1:**
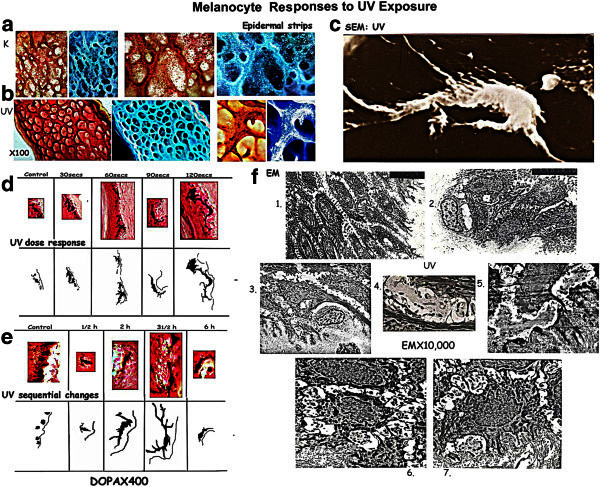
**Melanocyte Responses to UV exposure.** [**a**] Epidermal strips: control: non dendritic melanocytes. [**b**] UV exposure: Dendritic melanocytes in G_2_ phase. [**c**] SEM: Dendritic melanocytes in 3D. [**d**]: Organ Cultures: UV dose response: Melanocyte dendricity increases with dose, maximum on 120 s UV exposure. [**e**]: Sequential changes: Dendricity increases sequentially on exposure to a pulse of 120 s UV, maximum being at 3½ h, the effect lasting for 3 h. [**f**]: Ultrastructure of epidermis: 1. Intercellular spaces between keratinocytes reach down to the basement membrane. 2/3. Melanocytes on the BM with surrounding keratinocytes. 3–7. UV exposure: Melanocyte dendritic processes extend into the intercellular spaces supported by desmosomes.

Whole skin Organ Cultures [OCs] and epidermal strips subjected to direct UV exposure showed no change in marginal melanocytes. When incubated with adriamycin, and arrested in the G_2_ phase, prominent dendricity is observed on exposure to UV.

#### Melanocyte UV dose response: [Figure 1d]

18 of the 25 biopsies showed photoresponsive melanocytes showing prominent dendricity, in response to increasing UV exposure - 90 of 125 MUs. OCs were harvested at 3 h after exposure when maximum photoresponse is observed [see sequential data]. 125 marginal MUs were assessed in each set. Controls show 15% dendricity ie in 19MU. With 30s UV dendricity is in 4% [5MU]. Thereafter the dendricity increases to 20% [25MU] with 60s UV, 73.3% [92MU] with 90s and 93.3% [117MU] with 120 s UV exposure. At 6 h after 120 s UV dendricity is 18% 23MU. Doses beyond 120 s caused disruption of the epidermis. Thus UV exposure in the G_2_ phase results in dendricity with the maximum response with 120 s exposure, the effect lasting for 3 h.

A one-way analysis of variance test showed a statistically significant difference in dendricity in marginal melanocytes when compared with controls on UV exposure (F = 30.81, p < 0.001). Planned comparisons using the Holm-Sidak method showed that there were no difference in dendricity in control cultures vs 30s while there was a significant increase in these values beyond 30s.

#### Melanocyte UV sequential changes: [Figure 1e]

Melanocyte photoresponse was seen in 19 of the 25 biopsies on exposure to 120 s UV in the G_2_ phase harvested to study sequential changes.

19 of the 25 biopsies showed photoresponse - 95 of 125 MU, melanocytes showing dendricity, in response to 120 s UV exposure. OCs were harvested at ½ h, 2 h, 3½ h, and 6 h after UV exposure. Controls show 31% dendricity, in 39MU. With ½ h UV dendricity is 6% [8MU]. Thereafter the dendricity increases to 37% [46MU] at 2 h UV, 67% [84MU] at 3 h. At 6 h after 120 s exposure dendricity is 16.7% [21MU]. Thus 120 s UV exposure in the G_2_ phase results in dendricity with the maximum response at 3 h, the effect lasting for 3 h beyond peak dendricity.

Ultrastructure (Figure 1f) reveals the details of dendritic processes extending into the intercellular spaces supported by desmosomes towards UV.

Melanocytes showed statistically significant dendricity on UV exposure as compared to controls (ANOVA, F = 8.21, p < 0.001). Multiple comparisons using the Holm-Sidak method showed that there were no significant differences dendricity in melanocytes, 0.5 h after UV exposure. There was a significant increase in dendricity at 2 h and 3.5 h after UV exposure compared to that in control cultures. In contrast, there was a significant decrease in dendricity 6 h after UV as compared to controls.

Epidermal Strips: (Figure 1a-b). The composite figure brings out the photoresponse with dendritic melanocytes extending into the depigmented zone.

Ultrastructure: (Figure 1a&f) SEM and EM studies outline the 3D status of the melanocytes and the growth of the dendrites in response to UV exposure.

#### Response to IR exposure: [Figure 2]

**Figure 2:**
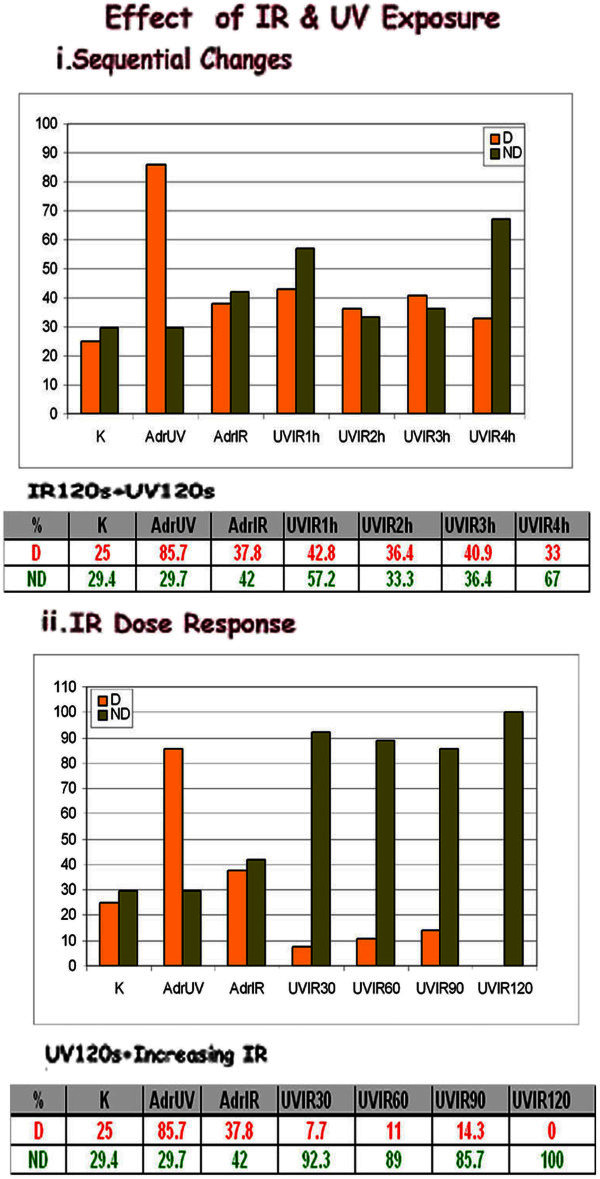
**i: Melanocyte UVvsIR sequential changes: UV induced dendricity shows a flat curve on combined UV/IR exposure.** ii: Melanocyte UVvsIR dose response: UV induced dendricity is abolished with increase in nondendricity, on increasing doses of IR. Non-dendricity increases to 92.3% with 30s IR, and 100% on 120 s IR, following an initial exposure to 120 s UV.

Melanocyte changes on IR and UV exposure in G_2_ phase were compared. Dendricity increases to 85.7% on UV exposure while IR shows 37.5% from control values of 25%. Non- dendricity does not change with UV but increases to 42% on IR from a control of 29.4%.

### Melanocyte UVvsIR sequential changes: [Figure 2i]

Dendricity shows a flat curve on combined UV/IR exposure with a decline to 42.8% from 85.7% on UV in the first hour, to 36.4% at 2 h, 40.9% at 3 h and 33% at 4 h. Thus IR + UV results in a combined effect of both wavelengths.

Multiple comparisons using the Holm-Sidak method showed that there were no significant differences in dendricity in melanocytes, after UV/IR exposure at each interval as compared to controls.

### Melanocyte UVvsIR dose response: [Figure 2ii]

When increasing doses of IR are given after 120 s UV exposure dendricity is abolished while there is a gradual increase in non-dendricity to 92.3% with 30s IR, 89% with 60s, 85.7% with 90s, which reaches 100% on 120 s IR following UV. Thus an initial UV exposure in the G_2_ phase is required for IR effect on melanocyte dendricity.

A one-way analysis of variance test showed a statistically significant difference in non-dendricity in marginal melanocytes on varied doses of IR on UV exposure (F = 30.81, p < 0.001). Planned comparisons using the Holm-Sidak method showed that there was no difference in non-dendricity in control cultures vs 30s while there was a significant increase in these values beyond 30s.

### Melanocyte functions on UV exposure: [Figure 3]

**Figure 3:**
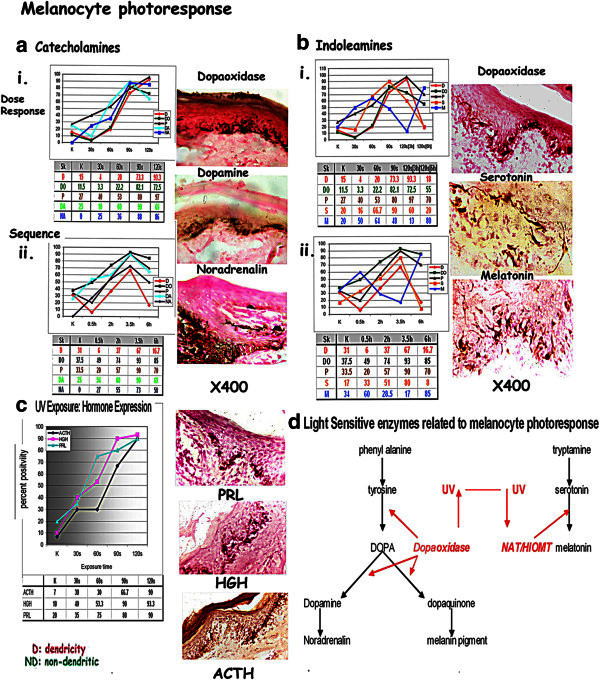
**Melanocyte functions on UV exposure: [a] i-ii:*Catecholamines:*Catecholoxidase, pigment transfer, dopamine, and noradrenalin: increase with increasing dose of UV and sequentially on exposure to 120 s UV [b] i-ii:*Indoleamines*: UV dose response:** Serotonin positivity increases with the dose of UV and sequentially to a 120 s pulse of UV to a peak at 3 h returning to control levels after 6 h. Melatonin decreases with increasing exposures of UV to13% after 120 s UV at 3 h followed by a rise to 85%, at 6 h incubation. [**c**]: *Hormone expression*: The melanocyte expresses ACTH: 80%, HGH: 85% and PRL: 93% 3 h after exposure to 120 s UV [**d**] Diagram depicting light sensitive enzymes NAT/HIOMT inhibited by light and catecholoxidase activated by light.

Serial sections were examined for biogenic amine activity, catecholamines and indoleamines, in OCs exposed to UV, both as dose and sequential effects.

### Catecholamines: [Figure 3a]

Serial sections were stained for dopaoxidase positivity using both tyrosine as well as dopa as substrates [giving identical results], pigment transfer, and by immunohistochemistry using mAb against tyrosine hydroxylase, dopamine and nor adrenalin.

### UV dose response: [Figure 3ai]

Dopaoxidase activity increases with increasing dose of UV concurrent with dendricity: control OCs show 11.5% [14MU] positivity; with 30s UV exposure there is a fall in activity to 3.3% [4MU]. Thereafter positivity increases to 22.2% [28MU] after 60s exposure; 82.1% [103MU] after 90s; and 72.5% [91MU] on 120 s exposure; on further dark incubation to 55% [69MU] at 6 h.

Pigment transfer: A similar trend is seen with pigment transfer to the keratinocytes of the MU: 27% [34MU] in controls; 40% [50MU] after 30s exposure 53% [66MU] after 60s; 80% [100MU] after 90s: and 97% [121MU] after 120 s. 6 h after 120 s pigment donation reduced to 70% [88MU].

Dopamine positivity shows an increase with increasing UV exposure; thus controls show 25% [31MU] while 30s exposure shows 10% [13MU]. On 60s exposure positivity increases to 60% [75MU]; and thence to 90% [112MU] on 90s exposure and 65% [81MU] after 120 s exposure.

Noradrenalin: The control shows no activity being 0%; at 30s exposure 25% [31MU] positivity is observed; which increases to 36% [45MU] on 60s exposure; 88% [110MU] on 90s; 86% [108MU] on 120 s exposure.

A one-way analysis of variance test showed that there was a statistically significant difference in catecholamine positivity in marginal melanocytes when controls were compared with UV exposure (F = 30.81, p < 0.001). Planned comparisons using the Holm-Sidak method showed that there were no differences in catecholamine positive melanocytes in control cultures and at 30s UV while there was a significant increase in these values beyond 30s UV.

### Catecholamines: UV sequential changes [Figure 3aii]

Dopaoxidase positivity in the marginal melanocytes in controls is 37.5% [47MU]; 49% [61MU] after ½ h followed by 74% [93MU] at 2 h and 93% [116MU] after 3 h incubation. Further incubation to 6 h shows a decrease to 85% [106MU].

Pigment transfer into keratinocytes in the marginal MUs follows the enzyme levels to a peak at 3 h with 90% [112MU] the lowest being at ½ h [20%: 25MU]. Controls show pigment transfer in 33.5% [42MU]; 57% [71MU] at 2 h and 70% [88MU] after 6 h.

Both Dopamine and Noradrenalin positivity is observed correlating with enzyme activity.

Dopamine activity shows a gradual increase from control values of 25% [31MU] to peak at 3 h: 90% [113MU]; followed by: 54% [68MU] at ½ h; 60% [75MU] at 2 h and 65% [81MU] at 6 h.

Noradrenalin activity follows the same trend, peak activity being at 3 h : 73% [91MU]. The controls show no activity, 27% [34MU] positivity being observed at ½ h increasing to 55% [69MU] at 2 h. Positivity declines to 50% [63MU] after 6 h incubation.

There was a statistically significant change in catecholamine positive melanocytes in the experimental cultures compared to that in controls (ANOVA, F = 8.21, p < 0.001). Multiple comparisons using the Holm-Sidak method showed that there were no significant differences between catecholamine levels in the melanocytes, 0.5 h after UV exposure. There was a significant increase in catecholamine positivity at 2 h and 3.5 h after UV exposure compared to that in control cultures. In contrast, there was a significant decrease in catecholamine levels in the melanocytes 6 h after UV treatment compared to that in controls.

Thus there is a gradual increase in dendricity, the triphasic dopaoxidase as well as pigment transfer on exposure to increasing levels of UV, and sequentially to a pulse of 120 s of UV, the peak being reached 3 h after exposure during the G_2_ phase. This is associated with a similar increase in the catecholamines, dopamine and noradrenalin. Thus the melanocyte photoresponse entails activation of the triphasic enzyme, which is dose related, with the production of pigment as well as catecholamines. This activation is not seen on dark incubation.

### Indoleamines: [Figure 3b]

Tyramine has been used as substrate to assess monoaminooxidase activity for evidence of indoleamines metabolism. Photoresponsive dendritic melanocytes show positivity for MAO indicating the presence of indoleamines activity. Serial sections were stained for indoleamines using mAb against serotonin as well as melatonin to study the pattern of indoleamines activity on UV exposure.

### UV dose response: [Figure 3bi]

Serotonin [Ser]: Serotonin positivity increases with the dose of UV to a peak after 90s of UV with 90% [112MU] after an initial fall to 16% after 30s UV exposure from 20% [25MU] control value; positivity is 66.7% [83MU] after 60s exposure and 60% [75MU] on 120 s exposure. Positivity returns to control levels after 6 h.

Melatonin [Mlt] decreases with increasing exposures of UV after an initial increase along with serotonin positivity to 50% [63MU] after 30s and 64% [80MU] after 60s UV exposure from 20% [25MU] control values. Thereafter Mlt positivity declines to 48% [60MU] after 90s exposure and 13% [16MU] after 120 s UV exposure. Peak levels of 80% [100MU] are seen after a further 3 hours incubation in the dark.

A one-way analysis of variance test showed that there was a statistically significant difference in indoleamine positivity in marginal melanocytes when controls were compared with treated cultures (F = 4.80, p = 0.004). This difference is due to the increase in SER and reciprocal decrease in MLT in melanocytes exposed to UV light at 90s and 120 s (3 h) compared to that in control (post-hoc tests performed using the Holm-Sidak method). There was no significant difference in SER levels in melanocytes in controls vs 30s, 60s and 120 s (6 h) UV. MLT is significantly higher at 120 s (6 h). Thus there was an increase in SER and decrease in MLT levels in melanocytes by 90s and a decrease of SER and an increase of MLT in 120 s (6 h).

### Indoleamines: UV sequential changes [Figure 3bii]

Serotonin positivity shows increases from control values of 17% [21MU] to peak at 3 h: 80% [100MU]; followed by: 33% [41MU] at ½ h; 51% [64MU] at 2 h and decreases to 8% [10MU] at 6 h.

Melatonin activity follows the opposite trend, lowest activity being at 3 h: 17% [21MU]. The controls show 34% [43MU], 60% [75MU] positivity being observed at ½ h decreasing to 28.5% [36MU]; at 2 h. Positivity increases to 85% [106MU] after dark 6 h incubation.

There was a statistically significant change in indoleamine positive melanocytes in the experimental cultures compared to that in controls (Kruskal-Wallis ANOVA on ranks, H = 9.886, p = 0.042). Multiple post-hoc comparisons using the Tukey test showed that there were no significant differences between SER positivity in controls vs 0.5 h, 2 h or 6 h after UV exposure and in levels of MLT after 0.5 h, 2 h, and 3 h. However, there was a significant increase in SER positivity in the melanocytes in K and 0.5 h vs 3.5 h after UV exposure. MLT shows significant decrease at 3.5 h and increase at 6 h compared to K and 0.5 h. Thus SER and MLT show reciprocal activity SER increasing on light exposure, while melatonin increases on dark.

From the above trends, it is seen that UV exposure initiates indoleamine production in the marginal melanocytes in G_2_ phase, to include both Ser and Mlt indicating the activation of the light sensitive enzymes NAT and HIOMT which convert Ser into Mlt at ½ h and on 30s exposure. As UV exposure increases Mlt levels fall while Ser increases due to the inhibition of the enzymes which convert serotonin to Mlt. Ser shows peak activity 3 h after UV exposure during the photoresponse to start declining in the next 3 h. Mlt on the other hand increases with decreasing UV indicating the activation of light sensitive enzymes to reach a peak 3 h after photoresponse is abolished.

### Hormone expression: [Figure 3c]

At the peak photoresponse at 3 h after exposure to 120 s UV, the melanocyte expresses ACTH: 80%, HGH: 85% and PRL: 93% as observed on monoclonal staining in both the groups.

On staining for ACTH expression with a mAb a graded increase in positivity is observed from 6.7% [control] to 30%, 30%, 66.7%, 90% on 30s, 60s, 90s and 120s UV, indicates expression of ACTH by the melanocytes on G_2_-phase UV exposure. Since this is part of the POMC molecule, POMC is expressed by the melanocytes.

The photoresponsive marginal melanocytes show increasing expression of PRL and HGH correlating with increasing UV exposure.

### Hair follicles

Melanocytes are poorly dendritic within the anagen matrix of 197 follicles present within 125 control and dark-incubated cultures. On UV exposure, 93.5% [182/194] of anagen follicles, present within 92 photoresponsive OCs, show highly dendritic melanocytes, the dendrites extending towards the hair shaft. They show all features of melanocytes UV responsiveness - positivity for biogenic amines, and hormones as observed in epidermal melanocytes.

### Photoperiod measurements: [Figure 4a-c]

**Figure 4:**
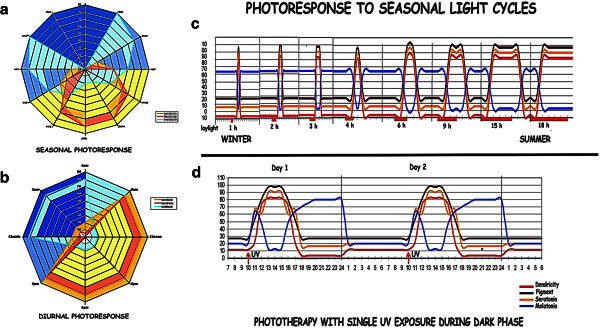
**Response to light cycles:** Dendricity, serotonin, and melatonin during [**a**] seasonal cycles and [**b**] the diurnal photophase. [**c**] Depicts the phase response curves, near the poles, measuring length of the photophase, with the serotonin/melatonin flux, during transit from winter, with short curves to summer with lengthening curves. [**d**] Correction of the serotonin/melatonin rhythm by phototherapy during long dark phase in winter.

The above experiments when viewed together simulate the reaction of the melanocytes to the daily/annual light cycles. The dose response data simulate the increasing light during sunrise and decreasing light during sunset, while the sequential data indicate the time taken for maximum response on light exposure. When applied to the circadian, if the sunrise is at 6 am the peak light is achieved between 8 am to 9 am, serotonin, dendricity and expression of other neurotransmitters and neurohormones in the active melanocytes is reached at 11 am to 12 noon and is maintained during the photoperiod. If sunset is at 6 pm, the activity continues till 8 pm to 9 pm, 3 h after last light, after which the Ser levels decline (Figure 4a-c). The photosensitive enzymes N-acetyl transferase [NAT] and hydroxyindole-o-methyl transferase [HIOMT] are activated converting Ser to Mlt to peak in next 3 h, that is at 12 midnight. The Mlt positivity is maintained till sunrise to decline to a minimal level 3 h after light exposure by the inactivation of NAT and HIOMT by light. This reciprocal activity of Ser and Mlt measures a photoperiod of 12 hours.

Similarly, in polar regions with large variations in light cycles the Ser/Mlt flux measures the photoperiod which increases during summer and decreases during winter. In the extreme north there are periods when no sunlight is presented. With absence of UV stimulation the Ser/Mlt production does not occur. This can lead to sleep disturbances and winter depression. Phototherapy with whole body irradiation corrects the balance as indicated in the figure. The imbalance is due to a complete depletion of serotonin production by the melanocytes as well as the other systems related to light entrainment (Figure 4d).

### Evidence of an organized system of photoreception in the skin

The dermatomic distribution of: photoactive melanocytes, the pigmentary disorders - vitiligo, melanosis, leprosy, and the melanocyte/keratinocyte tumors - melanomas, basal cell/skin adnexal and seborrheic keratosis has been assessed to study the organization of the system. Further, evidence of embryonal migration of active melanocytes has been studied by analyzing the pattern of pigmentation in a colony of guinea pigs.

### Melanocyte photosensory system [Figure 5]

**Figure 5:**
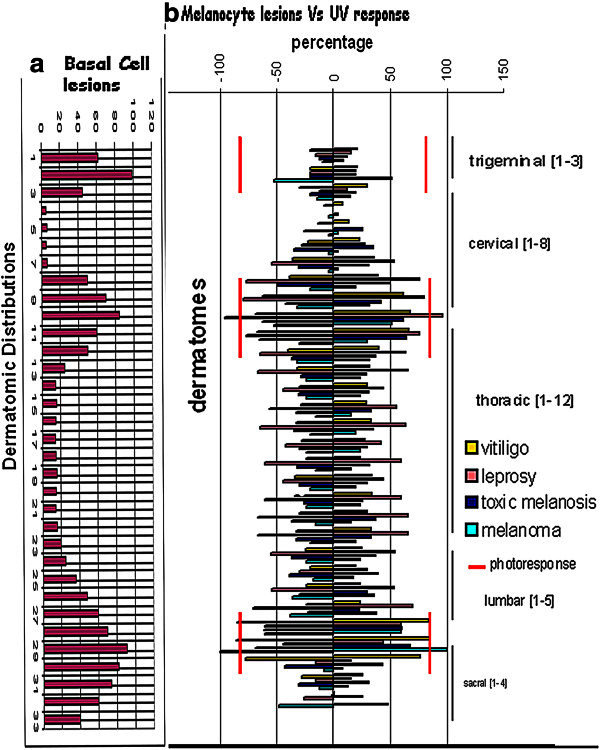
**Dermatomic maps to show favoured distributions of various lesions.** [**a**] Basal Cell/melanocyte lesions; [**b**] Composite map of photoresponsive melanocytes; vitiligo, leprosy, melanosis, melanomas; showing preferential distribution in the trigeminal, brachial and lumbosacral distributions.

#### Dermatomic distribution of photoactive melanocytes: [Additional file 1: Table S1]

Of the total 534 OCs of 356 cases exposed to a pulse of UV, 67% showed photoresponse while 33% were negative. On mapping and area wise analysis there is 81.5% photoresponse in the trigeminal area [44 of 54 OC], 79% along the brachial [75 of 95 OCs] and 78% along the lumbosacral dermatomes [212 of 273 OCs], the response is 27.3% in the cervical [12 of 44 OCs] and 22% in the thoracic regions [15 of 68 OCs]. Thus an actively photoresponsive population of melanocytes is demarcated in the trigeminal, brachial and lumbar dermatomic distributions.

#### Reactive melanocyte populations: [Figure 5b]

A detailed map of lesions, were made on cyclostyled outlines in 297 cases of vitiligo, 100 cases of melanosis, 142 cases of leprosy (Singh et al., [Bibr CR20][Bibr CR21]) and 135 cases of melanomas. The dermatomic distribution in the total number of cases was plotted as a percentage to study the pattern. Bilateral symmetry of lesions was also recorded.

##### Depigmentation

On an analysis of 297 cases of vitiligo bilateral symmetry was noted in 235 cases (79%). In 5 of these cases the distribution is along the area of supply of the dorsal branch of the dorsal root. On detailed mapping of lesions, the distribution is 36% in the trigeminal area, 15% along C_1_ to C_3_, 52% along C_4_ to T_1_, 27% along T_2_ to L_3_, and 82% from L_4_ to S_1,_ the areas prominently affected being in the trigeminal, brachial and lumbosacral distributions.

##### Hyperpigmentation

In 100 cases of Hypermelanosis the maximum in the trigeminal region is 20%, cervical_1-4_ is 18%, brachial plexus is 65%, thoracic region is 31%,lumbar plexus is 68%. The maximum distribution is thus in the brachial and lumbosacral distributions.

##### Hypopigmentation

In 142 leprosy cases there is striking bilateral symmetry and a similar dermatomic distribution. Lesions are seen in 20% in the trigeminal area, 2% from C_1_ to C_3_, in 77.7% from C_4_ to T_1_, 27% from T_2_ to L_3_, and 57% from L_4_ to S_1_. This distribution covers the entire spectrum of leprosy.

##### Proliferation

On an analysis of 135 cases of melanomas the distribution is 30.5% in the trigeminal area, 12.5% along C_4_ to T_1_, 6% along T_2_ to L_3_, and 51% from L_4_ to S_1,_ the areas prominently affected being in the trigeminal, brachial and lumbosacral distributions.

The location of reactive populations of melanocytes coincides with the areas of prominent photoresponsive suggesting a well organized pigmentary system within the skin.

#### Reactive keratinocyte populations: [Figure 5a]

A study of basal cell carcinomas skin adnexal tumors and lesions involving both melanocytes and keratinocytes ie pigmented basal cell carcinomas, seborrheic keratosis, lichen planus, and psoriasis, making a total of 442 lesions show a similar distribution pattern of dermatomic distribution: 42% in the trigeminal, 21.3% brachial, 20.7% lumbosacral, 11.3% thoracic and 2.3% in the neck, the largest number being in the trigeminal, brachial and the lumbosacral areas. These features indicate that apart from melanocytes the interacting keratinocytes are specific to these dermatomic areas as well.

#### Melanocyte migration from the neural crest: [Figure 6]

**Figure 6:**
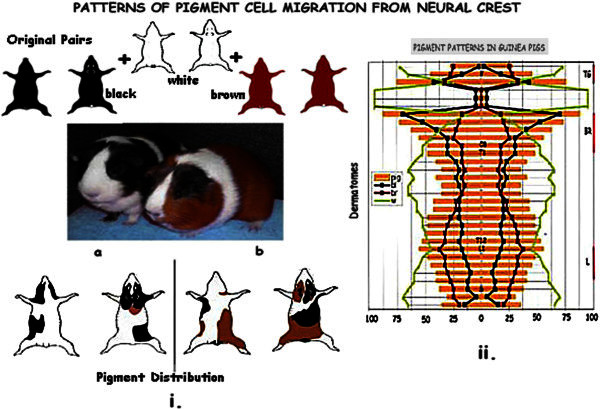
**Melanocyte migration in guinea pigs derived from i.** White, brown and black progenitors. **ii**. Pigment, indicating melanocyte migration, preferentially involve the trigeminal, brachial and lumbosacral distributions reflecting the active zones seen in the human material.

A colony of guinea pig bred from white, brown and black pairs was assessed to study the migration of pigment cells in relation to dermatomes. White areas do not show any melanocytes in the hair follicles and the epidermis as compared with brown and black patches. Thus the pattern of pigmentation represents the movement of the neural crest precursors of melanocytes to the epidermis.

### Pigment distribution in guinea pigs

Of the 285 guinea pigs 43 showed uniform colouring, 17 were brown, 16 black and 10 were totally white. 85 showed patches of all three colours involving the entire axis. These were analysed for dermatomic distribution. The trigeminal, brachial and lumbar distribution shows prominent pigment while thoracic show [T_2-12_] and cervical C_1_ to C_3_, show predominant white patches. Trigeminal shows a peak of 75% in V3, brachial a peak of 87.5% in C4, and lumbar a peak of 55%. Cervical 1–3 show 5% pigment and 95% white, while sacral area has a maximum of 47.5% pigment. Thus there is a preferential clustering and migration of pigment cells along specific dermatomes (Singh et al. [Bibr CR65][Bibr CR66]) in the trigeminal, brachial and lumbar areas. Pigment showed an overall bilateral symmetry but not in individual animals. 157 guinea pigs showed isolated or single coloured patches. Peak migration of pigment in these was seen in the trigegeminal, and brachial dermatomes and a proportion at the tail tip ie caudal 1–2, lumbosacral being spared.

## Discussion

Embryologically, the skin and the nervous system have a common origin from the ectoderm. The neuroectoderm is the first organ system to differentiate under the inductive influence of the primary inducer, Hensen’s node [HN], which becomes active at the centre of the embryonic disc. The inductive load giving rise to the complicated brain is six times that of the posterior half. The HN recedes at three times the speed in the posterior half of the neural axis. The midpoint and posterior end are induced (Iyengar and Lal 1982, 1984, 1985; Iyengar [Bibr CR20][Bibr CR21]) twice and coincide with the brachial and lumbar plexuses.

The elevating neural folds give rise to the neural crest cells which undergo epithelial/mesenchymal transition and take on migratory characteristics (Duncker 1985; Weston 1970, 1971; Kalcheim and Le Douarin 1999; Le Douarin and Dupin 2003). The anterior extension of the medullary plate gives rise to the CNS component of the neural crest cells, during the formation of the forebrain (Gans and Northcutt, 1983). Other cells originate in the neural folds but remain within the surface ectoderm after neurulation. These areas of neuroepithelium within the surface ectoderm are termed as ectodermal placodes. Paired sensory organs are formed by a combination of the neural plate, neural crest and ectodermal placodal material. The sensory ganglia of the CNS, sensory neurons of the spinal cord forming the spinal ganglia are from the neural crest (Northcutt and Gans, 1983; Detwiler 1937).

The neural crest placodal system, with its extensive migratory ability is responsible for the development of new sensory organs and their highly variable distribution, as demonstrated by the gustatory (Le Douarin and Dupin 2003), lateral line and electroreceptor organs (Fessard 1974) or the sensory skin innervation (Kalcheim and Le Douarin, 1999). The melanocytes are photosensory neural crest cells which migrate extensively into the skin. The question arises as to whether they form an organized system or are randomly deployed in the skin.

### The melanocyte photosensory system

The melanocyte system has all the attributes of a photosensory neural crest/placodal system: a] there are identifiable epidermal domains and b] both neural crest and placodal cells, the interacting keratinocytes, form photo-receptors – hair follicles and melanin units.

### Photoreceptors: [Figure 7c]

**Figure 7:**
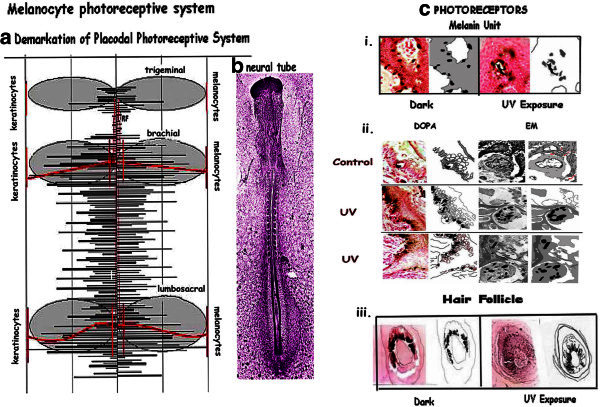
**Melanocyte photoreceptive system: a.** Dermatomic map outlining the placodal photoreceptive system shown as shaded areas. **b**. 40 h neural tube of chick embryo identifies areas of high inductive activity. **c**. Photoreceptors: i. Melanin Units: Non dendritic melanocytes on dark incubation and dendriticity on UV exposure. ii. Panel depicts melanin units on light and EM in controls and on UV exposure with dendrites extending towards the source of UV. iii. Hair follicle: Dark incubation shows non dendritic melanocytes in the hair bulb. UV exposure: Melanocytes show dendrites directed towards the shaft on UV exposure.

#### Hair follicle: [Figure 7ciii]

The formation of the hair follicle is reminiscent of the development of the electroreceptor organ in the fish, where the ectodermal placode sinks to the dermis to connect with the neural crest cell, as in the electroreceptor organs. The hair follicles form tactile organs, the vibrissae in many animals and have a rich innervation.

In man and animals responding to seasonal changes in diurnal photoperiod, the organisation of the hair follicle makes it suitable for photoreception during anagen phase (Lever and Schaumberg-Lever 1993). The melanocytes are present deep within the hair bulb, surrounded by the basaloid matrix cells in contact with the basement membrane. The matrix cells form a compact and compressed column of ‘ghost’ cells resembling the cells seen within the plant stems (Mandoli and Briggs [Bibr CR50][Bibr CR51]) which transmit light. Melanocytes, show directional dendritic extension, on UV exposure as light gets focused by the cells of the hair shaft functioning as a fibreoptic devise (Iyengar 1997a, 1998d). The melanocytes form the neural crest component, while the interacting keratinocytes represent the placodal cells. In animals with a thick coat the hair follicle melanocytes respond to light, the epidermis being covered by hair. In the weasel and the polar fox (Rust and Meyer 1969), this response is evidenced by seasonal coat color changes. In man, the epidermal melanin unit takes over most of the response.

The above is not universal, the mechanism of photoresponse varies in different species. Animals with a wave-like hair cycle, have no follicular melanocytes, since the hair between the periods of moulting remains in telogen. (Paus et al. 1990; Slominski et al. 1994; Müller-Röver et al. 2001). Melanocytic stem cells are present in the bulge region of the hair follicle throughout the hair cycle (Nishimura et al. 2002). These may be involved in the induction of melanomas (Gomez Garcia et al. 2010).

#### Melanin units: [Figure 7ci,cii]

The melanin unit consists of a melanocyte resting on the epidermal basement membrane surrounded by 2 to 3 layers of approximately 36 keratinocytes forming a 3D complex. The intercellular spaces between the keratinocytes open on to the space surrounding the melanocyte as can be seen on EM. On UV exposure in G_2_phase, dendrites extend through the intercellular spaces between the keratinocytes, using the desmosomes as scaffolding seen in the EM sections.

Keratinocytes within the epidermis can be identified as the placodal cells since the melanocyte migrates to contact them to form a three dimensional melanin unit (Lever and Schaumberg-Lever 1993). On UV exposure the melanocyte becomes dendritic and transfers neurotransmitters and hormones expressed during this response (Iyengar [Bibr CR26][Bibr CR27], [Bibr CR28][Bibr CR29][Bibr CR42]). The keratinocyte nuclei are protected from the direct effects of UV by the transfer of melanosomes from the melanocytes to form nuclear caps (Iyengar 1996b, 1998e). Transfer of PCNA from the melanocytes helps in the repair of any DNA damage (Iyengar 1998e). As observed earlier, dendrites extend in the direction of UV source through the intercellular spaces spanned by the desmosomes. The manner in which dendrites extend indicates that these intercellular spaces serve as fiber-optic channels for UV. Thus the keratinocytes of the MU are organised to focus light on to the melanocyte for activation. The interesting feature is that unlike the hair follicle, the MU shows high plasticity with the melanocyte moving along the basement membrane to contact new areas of keratinocytes during repigmentation, as in vitiligo.

### Functional aspects: [Figure 3]

#### Melanocyte photoresponse

In the pineal gland, melatonin is produced from serotonin during the dark phase by the action of the enzymes NAT and HIOMT (Underwood, 1989Gaudet et al. 1993). These enzymes are inhibited, on exposure to light, with the accumulation of serotonin (Armstrong, 1989). Melanocytes have all the components for active tryptophan metabolism, including tryptphan hydroxylase, tyraminoxidase, NAT and HIOMT. Marginal zone melanocytes are negative for indoleamines and enzymes in vitiligo, but can be activated on UV exposure when synchronized in G_2_ phase (Iyengar, 1994, 1998a, b) Since the photoresponsive melanocytes are positive for both serotonin and melatonin, a similar photo inhibitor activity is possible within them, if indoleamines are being metabolised by them.

Melatonin is known to inhibit melanin production (Slominski et al. 1989; Slominski and Pruski, 1993a). This is well illustrated when the melatonin positivity is compared with pigment donation. The pigmentation is inversely proportionate to melatonin positivity. This is accompanied by poorly dendritic melanocytes.

Early studies have shown that melatonin is involved in increased mitotic activity during the dark phase in diverse cell types [Lerner et al., 1958; Armstrong, 1989. In contrast several recent studies suggest that melatonin is one of the factors causing melanocyte destruction [Slominski and Pruski 1993a; Slominski et al. 1996, leading to depigmentation.

Melanocytes are photoresponsive cells as evidenced in animals which show coat color changes in response to large variations in day-night cycles. The marginal melanocytes in vitiligo have been used as the model in this study, since these cells undergo active repigmentation and depigmentation.

Melatonin lowers the dendricity and pigment production but enhances the cell number, during dark incubation, following exposure to a pulse of UV (Iyengar 2000). These observations emphasise the fact that therapeutic use of melatonin for melanomas and other cancers has to be carefully assessed [Slominski and Pruski 1993a; Slominski et al. 1993, Slominski and Pawelek 1998]. It has also been shown in lower animals that melatonin is involved in regeneration [Morita and Best 1993.

When these results are juxtaposed on the pigment changes in animals, exposed to large variations in the annual circadian [Slominski and Pruski 1993a, the relevance to coat color changes is clear. Near the polar regions, several days of complete darkness occur in winter, followed by very short spells of daylight as simulated by the above experiments. Melatonin from the pineal accumulates during the dark period (winter), in the circulation.

The melanocyte network reads length of the photoperiod through the flux in the indoleamine levels. This is accompanied by a diurnal (Figure 4a-b) or an annual flux in the hormone and neurotransmitter levels. (Figure 4c). The tyrosine metabolism is inactive to result in the white winter coat. In spring, the short periods of daylight, function like a short pulse of UV, resulting in melanocyte proliferation in the presence of high melatonin which repopulates the skin and hair follicles (Iyengar 2000). As the day length increases, light sensitive enzymes are inhibited and the serotonin-melatonin flux is re-established in melanocytes [Iyengar, [Bibr CR32][Bibr CR33] with increasing G_2_-phase sojourn of the melanocytes. This is followed by repigmentation and increase in serotonin [Iyengar, [Bibr CR26][Bibr CR27]. As the days lengthen, melanocytes are arrested in the G_2_-phase during the photophase.

Serotonin levels increase and melatonin goes down and the coat color becomes black or brown, since the catecholamines and pigmentation are associated with the photoresponse, while the white hair of the winter coat is shed (Rust 1965; Altmeyer and Holzman 1985) (Figure 3). As winter approaches the day length shortens short pulses of UV exposure and decreasing levels of serotonin with increasing melatonin, to ultimately result in hibernation. When the serotonin touches zero, the melanocytes are traversed into G_1_ phase by melatonin to remain quiescent during winter.

During the photoresponse the triphasic enzyme, dopaoxidase is activated to produce pigment and catecholamines from tyrosine and the melanocytes express ACTH [a component of POMC], HGH, and PRL (Iyengar 1995b; Iyengar 1997b) with the setting in of estrus.

The melanocyte network expresses POMC (Stohr et al. 1985; Iyengar, [Bibr CR26][Bibr CR27], Iyengar et al. 1996c, Slominski et al. 1993b; Hunt et al., 1994; Wakamatsu et al., 1997; Tsatmali et al., 2000, 2002) and the hormone PRL during photoresponse to UV and is thus linked to the opioid system and regulation of gonadotropins. This is accompanied by a dose related increase in pigment donation. These features are also reflected by the association of coat color changes and the estrus cycle (Altmeyer and Holzman 1985; Rust and Meyer, 1969) in animals in association with the seasonal photoperiod.

Thus melanocyte photoresponse hinges on two sets of photosensitive enzymes, NAT and HIOMT related to indoleamines, and the triphasic dopaoxidase related to catecholamine (Figure 3).

### Melanocyte interactions

#### Neuro-endocrine function

The melanocyte lies on the epidermal basement membrane and interacts with the underlying dermal structures, including the rich vascular and the neural networks (Williams et al. 1995). The melanocyte-vascular interaction serves as a neuroendocrine function of this system, with the seasonal variation of hormone levels in animals and in man, when exposed to large variations in the day-night cycle (Lehninger et al. 1993).

### Neural interaction: [Figure 8]

**Figure 8:**
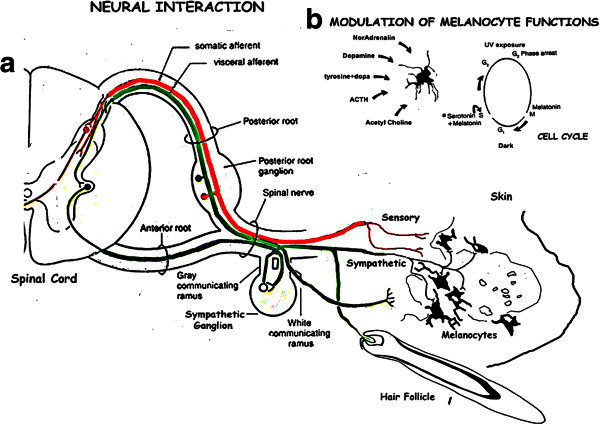
**Composite diagram showing the schema of interactions and modulation of melanocyte functions.** [**a**] Diagramatic representation of the sympathetic and sensory innervation, the possible pathways for entrainment of the seasonal photophase and light cycles through the melanocyte receptor complexes – melanin units and hair follicles, the cutaneous nerves being utilized as fiberoptic devises. [**b**] Modulation of the melanocyte cell cycle during the light and dark phases. Serotonin + Melatonin traverse and hold the melanocyte into G2 phase on UV exposure while Melatonin puts it through mitosis. NA, DA, ACTH and tyrosine + dopa enhance pigmentation while acetyl choline is inhibitory.

There is a fine network of sympathetic and sensory nerve twigs of neural crest in the reticular plexus lying just beneath the upper dermis. This network is very rich in the trigeminal, brachial and lumbosacral distribution. Fine fibres from this plexus enter the dermal papillae and hair bulb. In lower animals such as fish and reptiles the pigment cells are seen to be innervated by the sympathetic fibres (Whimster 1965).

Melanocyte functions and cell cycle are modulated by the rich dermal vascular and neural net works. A study of dendritic melanocytes at the margin of vitiligo, show a high enzyme activity which extends along the dendrites (Iyengar 1992b). These cells show biphasic melanogenic/adrenergic differentiation as is also observed in the amelanotic melanoma cell line HT-18 (Whimster 1965). The enzymes include tyrosine hydroxylase/dopa oxidase, dopamine oxidase along with the catecholamines, dopamine and noradrenaline activity. It was observed that the substrates of catecholamine metabolism modulate tyrosine metabolism, melanogenesis is enhanced by DOPA and tyrosine + DOPA, adrenalin and dopamine while cupric ions enhance catecholamine production as an autocrine feedback (Iyengar and Misra 1987). Melanocytes show membrane bound acetyl choline esterase (AChE) activity (Iyengar 1989). Acetylcholine (ACh) modulates pigmentation by inhibiting dopa oxidase activity in vitiligo during depigmentation, due to a decrease in melanocyte AChE, while during repigmentation esterase activity increases blocking thls inhibitory effect (Iyengar 1989). Skin biopsies from pigmentary lesions including melanocytic nevi and neurofibromas were also studied to assess neural dependence of epidermal melanocytes. Thus melanocyte functions are modulated by Ach, DA and NA in close association with dermal innervation as well as autocrine feedback by neurotransmitter substances produced by the melanocytes themselves, muscarinic acetylcholine receptor subtypes expressed in human skin melanocytes (Buchli et al. 2001; Plonka et al. 2009).

Several studies have substantiated these facts. Segmental-type vitiligo results from the dysfunction of sympathetic nerves in the affected area (Wu et al. 2000). Vital functions in keratinocytes and melanocytes are regulated by an autocrine adrenergic and cholinergic, intra/intercellular signal transduction network, in the human epidermis (Grando et al. 2006). The human skin serves as an extension of the neuronal network including both the adrenergic and cholinergic signal transduction pathways (Roosterman et al. 2006). Human keratinocytes and melanocytes synthesize and degrade acetylcholine (Kurzen and Schallreuter 2004). An interactive network between cutaneous nerves, the neuroendocrine axis, and the immune system has been established.

The intimate working relationship between the melanocyte and the underlying neural network can be observed in leprosy. Lepra bacilli are neurotropic and metabolise catecholamines (Yoshizumi and Asbury 1974). Lesions in leprosy coincide with areas showing photoactive melanocytes which produce catecholamines. The presence of bacilli in the nerve twigs in these areas indicates the uptake of catecholamines by the neural network from the epidermal melanocytes. Areas of depigmentation with loss of melanocyte photoactivity are associated with areas of anaesthaesia (Vemuri et al. 1995; Singh et al. 1983a). Thus the photoactive melanocytes interact with the sensory neural network by transferring catecholamines.

#### Communication with the central nervous system: [Figure 4d,8]

In man the effective treatment of SAD and MDS is by whole body irradiation with bright light showing a link between light exposure to the skin and CNS responses (Sugden 1989). This suggests that the melanocyte network transmits information about the environmental light cycles to the central nervous system by the circulation and the neural circuitary.

The catecholamine neurons in the dorsal root ganglion and the sympathetic chain are likely pathways, pigmentation indicating activation of the light sensitive dopaoxidase enzyme as is depicted in Figure 3d. Detailed scrutiny of the ultra structure of keratinocyte arrangement discloses an intricate network of intercellular spaces extending down to the BM in areas devoid of melanocytes. As discussed, since UV/light is transmitted through these spaces light can reach down to dermis with a rich network of nerve endings [Figure 1f (1–7)].

This study outlines the presence of a well defined melanocyte photosensory system in the skin with the well marked epidermal domains, an outcome of primary induction. As in the gustatory and other neural crest derived sensory systems, the pigmentary system is derived from a combination of neural crest and placodal elements which interact closely with the underlying neural net (Figure 7a). As in the gustatory system, the melanocyte photosensory system functions independent of the CNS, although it has a profound effect on the latter.

Traditionally photoreception is attributed to the eyes and the visual pathways, and to the pineal as the zeitgeber by monitoring the indoleamine levels related to the melatonin signal (Lloyd and Rossi 1992, Sugden 1989). Melanopsin (Provencio et al. 1998; 2000) in ganglion cells of the retina, initially isolated from the photosensitive dermal melanophores of *Xenopus laevis* react to the the environmental light/dark cycle. Melanopsin containing cells are intrinsically true photoreceptors (Bellingham and Foster 2002, Berson et al. 2002; 2010). They receive synaptic input and feed-back to dopaminergic amacrine cells (Vugler et al., 2007). Epidermal melanocytes replicate the photoresponses of melanopsin cells as observed in this study.

## Conclusion

Herbert 1989, enunciated the concept of neural systems required for photoperiodic time measurements. The melanocyte system measures the seasonal variations in the photoperiod by the same melatonin/serotonin switch. The environmental light cue is translated into chemical messages with a surge in neurotransmitters and neurohormones. Light/chemical messages transmitted to the CNS controls the sleep-waking cycles by acting on the arousal system of the reticular formation and LC. The identification of this photosensory system opens up new and exciting vistas pointing to a hitherto unexplored role played by the intricate colour patterns in living organisms, as a neurosensory system.

## Material and methods

### Experimental assessment of photoresponse

#### Photoresponsive melanocytes

##### Whole skin organ cultures [OC]

Whole skin organ culture was done on 356 random biopsies from the marginal zone of vitiligo to assess melanocyte photoresponse. Biopsies, 1.5 cm × 0.5 cm in size were taken to include both pigmented and non-pigmented areas. The tissues were transported in sterile MEM medium. These were cut into 8 bits each, 2 mm in width to include the marginal zone between the pigmented and vitiliginous zone under sterile conditions.

One bit from each biopsy [a] is immediately fixed in cold buffered formol-glutaraldehyde to serve as control. The rest were placed in micropetridishes 1 cm in diameter, containing 2000 μl MEM with 200 μg of adriamycin (Iyengar, 1992a) to synchronise melanocytes in the G_2_-phase and incubated at 37°C in the dark. The tissues were placed so that the epidermal surface was above the fluid level to simulate normal skin.

##### Photoresponses: [Table 1]

**Table 1:** **Schema: Grid showing tally of melanocytes counted in each experimental group**

UV dose 25 cases	K	30s	60s	90s	120s	T	UV Seq 25 cases	K	1/2 h	2 h	3.5 h	6 h	T	
OC	25	25	25	25	25	125	OC	25	25	25	25	25	125	250
MU	X5	X5	X5	X5	X5	X5	MU	X5	X5	X5	X5	X5	X5	X5
DO	125	125	125	125	125	625	DO	125	125	125	125	125	625	1250
TO	125	125	125	125	125	625	TO	125	125	125	125	125	625	1250
Pig	125	125	125	125	125	625	Pig	125	125	125	125	125	625	1250
DA	125	125	125	125	125	625	DA	125	125	125	125	125	625	1250
DAO	125	125	125	125	125	625	DAO	125	125	125	125	125	625	1250
NA	125	125	125	125	125	625	NA	125	125	125	125	125	625	1250
MAO	125	125	125	125	125	625	MAO	125	125	125	125	125	625	1250
S	125	125	125	125	125	625	S	125	125	125	125	125	625	1250
M	125	125	125	125	125	625	M	125	125	125	125	125	625	1250
ACTH	125	125	125	125	125	625	ACTH	125	125	125	125	125	625	1250
PRL	125	125	125	125	125	625	PRL	125	125	125	125	125	625	1250
HGH	125	125	125	125	125	625	HGH	125	125	125	125	125	625	1250
	1500	1500	1500	1500	1500	7500		1500	1500	1500	1500	1500	7500	15000
**UV vs IR 25 cases**	**K**	**30s**	**60s**	**90s**	**120s**	**T**	**UV + IR 25 cases**	**K**	**1/2 h**	**2 h**	**3.5 h**	**6 h**	**T**	
OC	25	25	25	25	25	125	OC	25	25	25	25	25	125	250
MU	X5	X5	X5	X5	X5	X5	MU	X5	X5	X5	X5	X5	X5	X5
DO	125	125	125	125	125	625	DO	125	125	125	125	125	625	1250
TO	125	125	125	125	125	625	TO	125	125	125	125	125	625	1250
Pig	125	125	125	125	125	625	Pig	125	125	125	125	125	625	1250
DA	125	125	125	125	125	625	DA	125	125	125	125	125	625	1250
DAO	125	125	125	125	125	625	DAO	125	125	125	125	125	625	1250
NA	125	125	125	125	125	625	NA	125	125	125	125	125	625	1250
MAO	125	125	125	125	125	625	MAO	125	125	125	125	125	625	1250
S	125	125	125	125	125	625	S	125	125	125	125	125	625	1250
M	125	125	125	125	125	625	M	125	125	125	125	125	625	1250
ACTH	125	125	125	125	125	625	ACTH	125	125	125	125	125	625	1250
PRL	125	125	125	125	125	625	PRL	125	125	125	125	125	625	1250
HGH	125	125	125	125	125	625	HGH	125	125	125	125	125	625	1250
	1500	1500	1500	1500	1500	7500		1500	1500	1500	1500	1500	7500	15000

*UV dose response:* [a] In 25 random biopsies from the above, one bit each was exposed to a pulse of 30s, 60s, 90s and 120 s of UV at 2 h of incubation [a total of 125 OCs]. The tissues were reincubated in the dark and were harvested at 3 h and one bit exposed to 120 s after 6 h of dark incubation after UV exposure.

*UV sequential changes:* [b] 4 bits were each exposed to a pulse of 120 s of UV in a second batch of 25 random biopsies from the above at 2 h of incubation. The tissues were reincubated in the dark. One tissue each was harvested at ½ h, 2 h, 3 h and 6 h to study the sequential changes on photoresponse [125 OCs].

*UV/IR combined response:* Two sets of experiments were conducted in 25 biopsies, 125 OCs in each group, to assess the dose response and sequential changes on UV/IR combined exposure. Whole skin organ cultures incubated with adriamycin were exposed to [a] 30s, 60s, 90s & 120 s IR ½ h after exposure to 120 s of UV to assess the dose response and [b] assessed for sequential changes ½ h, 2 h, 3½ h and 6 h after exposure to 120 s UV +120 s IR at 2 h of incubation in MEM with adriamycin.

UV exposure was done in a sterile, light tight hood, in atmospheric air to simulate normal sun exposure to the skin surface. A 15 watt flourescent tube emitting UV in the 280-400 nm range (peak emission at 280-340 nm and a peak at 254 nm; Phillips, Holland) was fixed in the hood. [Spectral output: 280-400 nm: Doses 30s:6.58X10^-7^ J; 60s:13.6X10^-7^ J; 90s:19.74X10^-7^ J; 120 s:26.32X10^-7^ J; Photon Energy: 3.3 eV].

IR exposure was done with a Phillips, Holland Bulb, HL 4307, 150 watts, Spectral output: 700-750 nm placed at the same distance as the UV tube. [Spectral output: 700-750 nm: Doses 30s:3.29X10^-7^ J; 60s:6.8X10^-7^ J; 90s:9.87X10^-7^ J; 120 s:13.16X10^-7^ J; Photon Energy: 1.7 eV]. The specimen were placed at a distance of 30 cm from UV/IR source (range 280–400 nm & 700-750 nm), and exposed to UV/IR after 2 h of dark incubation to allow the tissue to react with adriamycin. [Tables 2,3,4,5,6].

**Table 2:** **Table showing energy inputs of UVR exposure**

UVR Doses			
	Name	Wavelength	Photon Energy (eV)
	Ultraviolet	280 nm-380 nm	3.3 eV
	Visible	380 nm-700 nm	1.7 eV-3.3 eV
	Infrared	700 nm-750 nm	1.7 eV

**Table 3:** **UV dose and sequential doses**

UV	K	30s	60s	90s	120s
3.3 eV	K	6.58 × 10^-7^ J	13.6 × 10^-7^ J	19.74 × 10^-7^ J	26.32 × 10^-7^ J
	K	½ h	2 h	3½	6 h
UV 120 s	K	26.32 × 10^-7^ J	26.32 × 10^-7^ J	26.32 × 10^-7^ J	26.32 × 10^-7^ J

UV pulse 2 h after dark incubation in adriamycin.

**Table 4:** **Energy inputs with UVR exposures**

UV		K	30s	60s	90s	120s
3.3 eV	UV	K	6.58 × 10^-7^ J	13.6 × 10^-7^ J	19.74 × 10^-7^ J	26.32 × 10^-7^ J
IR	IR	K	3.29 × 10^-7^ J	6.8 × 10^-7^ J	9.87 × 10^-7^ J	13.16 × 10^-7^ J
1.7 eV						

UV/IR doses.

**Table 5:** **120s UV with graded IR exposures**

UV/IR	K	AdrUV	AdrIR	120sUV	120sUV	120sUV	120sUV	
		26.32 × 10^-7^ J	13.16 × 10^-7^ J	26.32 × 10^-7^ J	26.32 × 10^-7^ J	26.32 × 10^-7^ J	26.32 × 10^-7^ J	UV
		X120s	X120s	X30sIR	X60sIR	X90sIR	X120sIR	PLUS
				3.29 × 10^-7^ J	6.8 × 10^-7^ J	9.87 × 10^-7^ J	13.16 × 10^-7^ J	IR

UV pulse 2 h after dark incubation in adriamycin followed ½ h later by IR pulse.

**Table 6:** **Time sequence of combined UVR**

UV + IR	K	AdrUV	AdrIR	½ h	2 h	3½	6 h	
		26.32 × 10^-7^ J	13.16 × 10^-7^ J	13.16 × 10^-7^ J	13.16 × 10^-7^ J	13.16 × 10^-7^ J	13.16 × 10^-7^ J	IR
		X120s	X120s	X120s	X120s	X120s	X120s	PLUS
				26.32 × 10^-7^ J	26.32 × 10^-7^ J	26.32 × 10^-7^ J	26.32 × 10^-7^ J	UV
				X120s	X120s	X120s	X120s	

UV immediately followed by IR pulse 2 h after dark incubation in adriamycin.

After harvesting all specimens are fixed in cold buffered formolglutaraldehyde overnight, to prevent diffusion of enzymes and neurotransmitters, while retaining their activity. Formolglutaraldehyde is prepared in phosphate buffer (pH 7.2), with 10% formalin (4% formoldehyde) and 1% glutaraldehyde. 40 Serial, 5 μm thick frozen sections are cut on a Lipshaw cryotome at −25°C. Serial sections are stained for HE, dopa as substrate for dopaoxidase (Pearse AGE 1980) and the next section without substrate to assess pigment in all tissues.

Melanocyte morphology is assessed on dopa staining in marginal melanocytes and within the hair bulb. The number of melanocytes (a total of 1500 melanin units) showing dendricity at each exposure is depicted as percentage.

### Histochemistry and enzyme histochemistry

Silver stain for noradrenalin, and enzyme staining for dopaoxidase/tyrosinase with dopa/tyrosine as substrate; dopaminoxidase using dopamine and tyraminooxidase with tyramine as substrate (Pearse AGE 1980) in all tissues confirmed by immuno-staining.

Immunohistochemistry: was by the avidin biotin method (Mikel, 1994) using monoclonal antibodies (mAb) against each marker. The changes in marginal melanin units [MU] in each section (Fitzpatrick and Breathnach 1963) have been studied in the following, since this zone shows active depigmentation and repigmentation.

#### mAb Positivity

Melanocyte immuno-positivity was assessed in serial sections, by the avidin-biotin method, for the following markers, tyrosine hydroxylase [TH], tyrosinase [TO] dopaoxidase [DO] dopaminoxidase [DAO] dopamine [DA] & noradrenalin [NA], tyraminoxidase [MAO] [Dako Pat kits] Serotonin; Melatonin; [BioGenex Labs]:. Adrenocorticotropic Hormone [ACTH]; Prolactin [PRL]; Human Growth Hormone [hGH]: [Dako Pat kits] Counts of immuno-positive marginal melanocytes, for each UV exposure were done on 5 marginal melanin units in each section, for each of 12 markers [1250 melanocytes counted for each mAb]. The primary antibody was excluded to serve as a negative control, while mAb serotonin and melatonin served as positive controls for each other. In addition, suitable positive controls were used in each case.

### Epidermal strips

20 biopsies were subjected to epidermal strips which were subjected to the same pattern of UV exposure as the OCs with the strips immersed as above. Epidermal strips were prepared by immersing 2 mm wide bits from skin biopsies in NaBr solution [205 g NaBr in 1 L of distilled water] for ½ h under sterile conditions. The epidermis is to be carefullly stripped off with fine forceps and immersed, with in 2000 μl MEM containing 200 μg of adriamycin in micropetridishes, for dark incubation and UV exposure. The epidermal strips were whole mounted on slides with the dermal side up, subjected to enzyme staining for dopa. The strips were then whole mounted in DPX after dehydration.

Statistical analysis was done by ANOVA. Kruskal-Wallis One Way Analysis of Variance; All Pairwise Multiple Comparison Procedures [Tukey Test].

#### Evidence of an organized system of photoreception in the skin

### Dermatomic distributions

#### Photoresponse

In all 356 biopsies, one bit was incubated in the dark while one bit was exposed to a pulse of 120 s of UV at two hours of dark incubation. All the organ cultures were harvested at 5 h of incubation. The photoresponse was assessed as marginal zone melanocyte dendricity in 5 marginal MU on dopa staining. The percentage photoactivity at each dermatomic distribution is calculated that is trigeminal, cervical [C1-C4], brachial [C4-T1], thoracic [T2- T12] and lumbosacral [L1- S2].

#### Pigmentary disorders in man

A detailed map of lesions on cyclostyled outlines were made in 297 cases of vitiligo, 100 cases of melanosis, 165 cases of melanomas and 142 cases of leprosy. The dermatomic distribution in the total number of cases was plotted as a percentage to study the pattern. Overall bilateral symmetry of lesions was also recorded.

#### Reactive keratinocyte populations

Basal cell carcinomas, skin adnexal tumors and lesions involving both melanocytes and keratinocytes ie pigmented basal cell carcinomas, seborrheic keratosis, lichen planus, and psoriasis, making a total of 442 lesions, were similarly assessed for pattern of dermatomic distribution.

#### Assessment of melanocyte migration; pigment distribution in guinea pigs

Pigment distribution in 285 guinea pigs from an inbred colony having black, brown and white patches, represented along the entire neural axis was assessed. A detailed map of the pigment patches in individual animals was drawn on a cyclostyled outline to study the dermatomic distribution in each case. The total percentage of pigment along each dermatome was calculated to study the pattern of distribution.

All procedures were performed with the informed consent of patients for therapeutic purposes, in full compliance with the Helsinki Declaration.

## Electronic supplementary material

Additional file 1: Table S1: Table showing the organ cultures performed with those showing UV response. The total number of melanocytes counted and the percentage UV response in each dermatomic area is shown and depicted as a bar diagram. (JPEG 271 kb) (JPEG 272 KB)

## Abbreviations

[MU]: Melanin units
[SEM]: Scanning electron microscopy
[EM]: electron microscopy
[IR]: Infrared
[UV]: ultra violet
[DO]: Dopaoxidase
[DA]: Dopamine
[NA]: Noradrenalin
[Ser]: Serotonin
[Mlt]: Melatonin
[NAT]: N-acetyl transferase
[HIOMT]: hydroxyindole-o-methyl transferase
[ACTH]: Adrenocorticotropic Hormone
[POMC]: Pro-opio-melanocortin
[PRL]: Prolactin
[HGH]: Human Growth Hormone
[HN]: Hensen’s node
[Ach]: Acetylcholine
[SAD]: Seasonal affective disorder
[LC]: Locus Caeruleus.
