# Supplementary figures and images for: The melanocyte photosensory system in the human skin
Source: Springerplus. 2013 Apr 12;2:158. doi: 10.1186/2193-1801-2-158 (PMC3685707; doi:10.1186/2193-1801-2-158)

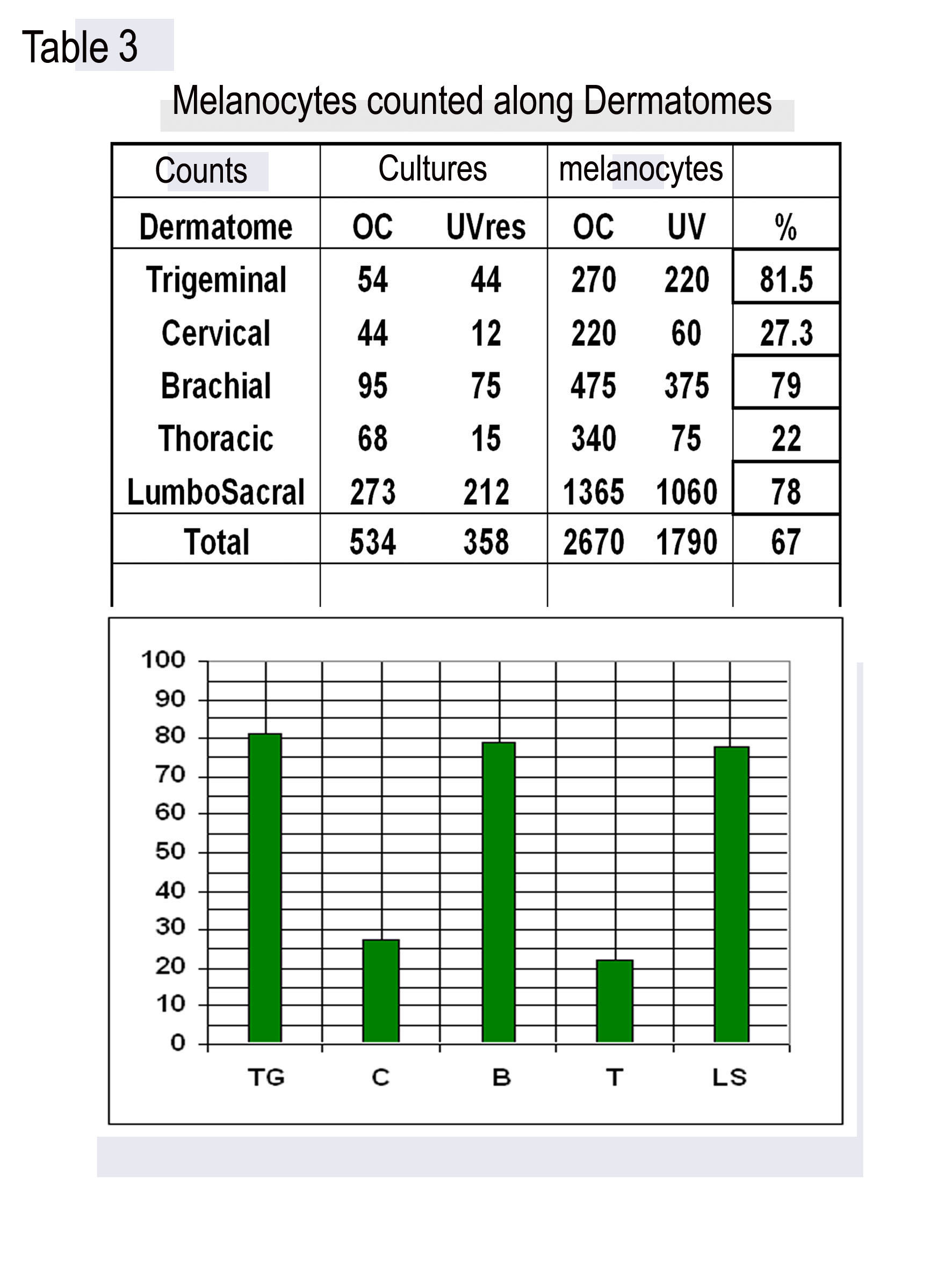

Supplement: Supplementary file 1 — Additional file 1: Table S1: Table showing the organ cultures performed with those showing UV response. The total number of melanocytes counted and the percentage UV response in each dermatomic area is shown and depicted as a bar diagram. (JPEG 271 kb) (JPEG 272 KB) [file 40064_2012_320_MOESM1_ESM.jpeg]
